# Modulation of the pharmacological effects of enzymatically-active PLA_2 _by BTL-2, an isolectin isolated from the *Bryothamnion triquetrum *red alga

**DOI:** 10.1186/1471-2091-9-16

**Published:** 2008-06-06

**Authors:** Simone CB Oliveira, Fabiana V Fonseca, Edson Antunes, Enilton A Camargo, Rafael P Morganti, Ricardo Aparício, Daniela O Toyama, Luís OS Beriam, Eudismar V Nunes, Benildo S Cavada, Celso S Nagano, Alexandre H Sampaio, Kyria S Nascimento, Marcos H Toyama

**Affiliations:** 1 Biochemistry Department, Biology Institute, UNICAMP, Campinas, São Paulo, Brazil; 2 Pharmacology Department, University of Medical Science, UNICAMP, Campinas, São Paulo, Brazil; 3 Chemistry Institute, UNICAMP, Campinas, São Paulo, Brazil; 4 University of Biological and EXaperimental Sciences, University of Mackenzie, São Paulo, São Paulo, Brazil; 5 Laboratory of Vegetal Bacteriology, Experimental Center of the Biological Institute, Campinas, São Paulo, Brazil; 6 Institute of Biomedicine of Valencia, IBV, Valencia, Spain; 7 Lab. of marine biochemistry -BioMar-Lab, Fishing Engeneer Department, Federal University of Ceará, Fortaleza, Ceará, Brazil; 8 UNESP, Litoral Paulista Campus, São Vicente, São Paulo, Brazil

## Abstract

**Background:**

An interaction between lectins from marine algae and PLA_2 _from rattlesnake was suggested some years ago. We, herein, studied the effects elicited by a small isolectin (BTL-2), isolated from *Bryothamnion triquetrum*, on the pharmacological and biological activities of a PLA_2 _isolated from rattlesnake venom (*Crotalus durissus cascavella*), to better understand the enzymatic and pharmacological mechanisms of the PLA_2 _and its complex.

**Results:**

This PLA_2 _consisted of 122 amino acids (approximate molecular mass of 14 kDa), its pI was estimated to be 8.3, and its amino acid sequence shared a high degree of similarity with that of other neurotoxic and enzymatically-active PLA_2_s. BTL-2 had a molecular mass estimated in approximately 9 kDa and was characterized as a basic protein. In addition, BTL-2 did not exhibit any enzymatic activity.

The PLA_2 _and BTL-2 formed a stable heterodimer with a molecular mass of approximately 24–26 kDa, estimated by molecular exclusion HPLC. In the presence of BTL-2, we observed a significant increase in PLA_2 _activity, 23% higher than that of PLA_2 _alone. BTL-2 demonstrated an inhibition of 98% in the growth of the Gram-positive bacterial strain, *Clavibacter michiganensis michiganensis *(Cmm), but only 9.8% inhibition of the Gram-negative bacterial strain, *Xanthomonas axonopodis *pv *passiflorae *(Xap). PLA_2 _decreased bacterial growth by 27.3% and 98.5% for Xap and Cmm, respectively, while incubating these two proteins with PLA_2_-BTL-2 inhibited their growths by 36.2% for Xap and 98.5% for Cmm.

PLA_2 _significantly induced platelet aggregation in washed platelets, whereas BTL-2 did not induce significant platelet aggregation in any assay. However, BTL-2 significantly inhibited platelet aggregation induced by PLA_2_. In addition, PLA_2 _exhibited strong oedematogenic activity, which was decreased in the presence of BTL-2. BTL-2 alone did not induce oedema and did not decrease or abolish the oedema induced by the 48/80 compound.

**Conclusion:**

The unexpected results observed for the PLA_2_-BTL-2 complex strongly suggest that the pharmacological activity of this PLA_2 _is not solely dependent on the presence of enzymatic activity, and that other pharmacological regions may also be involved. In addition, we describe for the first time an interaction between two different molecules, which form a stable complex with significant changes in their original biological action. This opens new possibilities for understanding the function and action of crude venom, an extremely complex mixture of different molecules.

## Background

Lectins are carbohydrate-binding proteins of non-immune origin found in a wide variety of living organisms that decipher glycocodes in the structure of glycans attached to soluble and integral cell membrane glycoconjugates [1]. Mechanisms for sugar recognition in microorganisms, plants, and animals are independently involved in several protein frameworks [2]. Protein-carbohydrate interactions play biological roles in many cellular processes, such as cell communication, host defence, fertilization, development, parasitic infection and tumour metastasis. Although, in many instances, their exact biological roles remain unknown, many lectins have been extensively studied as biochemical tools in biotechnology and biomedical research. Marine algal lectins, however, have been described at a low pace, since the first report on their haemagglutinating activity 40 years ago [3]. Marine organisms are recognized as rich sources of diverse and biologically-active molecules, many lectins from red and green marine algae have been isolated and characterised to date from more than 40 species. Amongst activities of potential therapeutic and diagnostic interest are the inhibition of fungal growth [4], induction and inhibition of human lymphocyte transformation [5], identification of methacillin-resistant *Streptococcus aureus *[6], induction of mitogenic activity [7], antibiotic activity against marine vibrios [8], endothelium-dependent relaxation of the rat aorta [9], inhibition of platelet aggregation [10], and as an anti-HIV protein [11].

In each family of lectins, the structure of their carbohydrate recognition domains (CRDs) is essentially conserved. Similar domains have been found in some phospholipase A_2 _(PLA_2_) receptors named M and N-type and play an important role in the pharmacological activity of PLA_2 _[12]. Phospholipases A_2 _(PLA_2_) (EC 3.1.1.4) from snake venom are very small proteins that catalyse the hydrolysis of glycerophospholipids at the sn-2 position in a Ca^+2^-dependent reaction, releasing lysophospholipids and fatty acids [13]. Snake PLA_2_s have many pharmacological effects, such as: neurotoxicity, myotoxicity, haemolytic activity, haemorrhagic and oedematogenic activities [13,14]. PLA_2_s that have lysine at the position 49 (Lys 49 or K49) [15] structurally keep the same motifs of PLA_2_s that have aspartate at the position 49 (Asp 49 or D49, catalytically active), but their enzymatic activity is lost. Despite this, these proteins display many biological activities, regardless of arachidonic acid release [16-18].

In 2000, Hori et al [19] developed the haemagglutinating activity assay, combining algal lectins (hypnin A) and PLA_2_s from the snakes, *Naja naja *and *Crotalus adamanteus*, and found an interaction between proteins, demonstrated by the inhibition of haemagglutination at a relatively low concentration [19]; it was suggested that this effect probably was dependent on a protein-recognition site present in the lectin surface where the PLA_2 _would bind. Thus, some algae lectin-like proteins may play other important biological functions, such as providing an alternative source of a novel class of PLA_2 _inhibitors. In this article, we investigate the effects induced by a new isolectin from the red marine alga, *Bryothamnion triquetrum*, on some of the biological activities of PLA_2 _and the interaction with catalytically-active PLA_2 _from snake venom.

## Results

**Figure 1:**
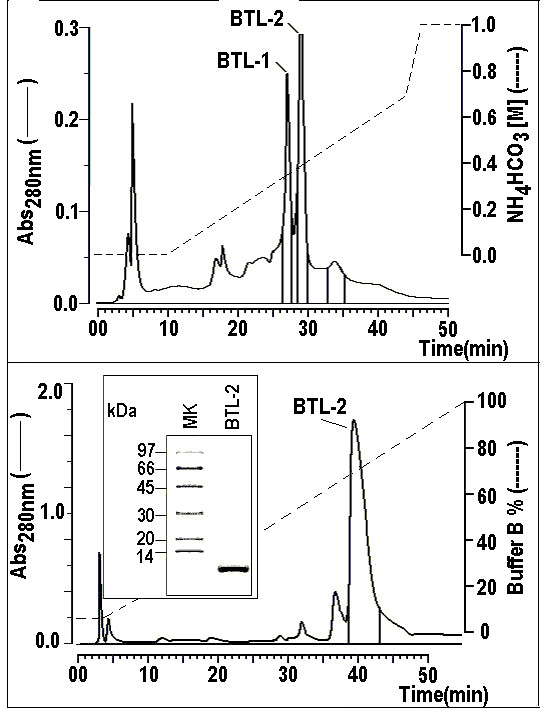
**1a) HPLC profile of BTL-2 using an ion exchange column, protein Pack SP 5PW (Waters).** Approximately 5 mg of the sample, obtained from the DEAE column, were dissolved in 0.05 M ammonium bicarbonate and applied onto the chromatographic column. The chromatography showed the presence of two main fractions containing haemagglutinating activity, named BTL-1 and BTL-2. The run was monitored at 280 nm and the elution was conducted using a discontinuous linear gradient of ammonium bicarbonate at a concentration of 1.0 M, pH 8.0, constant flow rate of 1 mL/min. 1b) The BTL-2 fraction was subjected to a final chromatography in a Reverse phase HPLC fractionation using a μ-Bondapack C18 column. The elution of samples was carried out using a linear gradient concentration of an aqueous solution of 66% acetonitrile and was monitored at 280 nm at a constant flow rate of 1 mL/min. Samples were previously dissolved in buffer A (0.1% TFA) that was used to equilibrate the column. 1c) Following the method [43], a discontinuous electrophoresis was performed with a final acrylamide concentration of 12% in the running gel. All the samples and the molecular markers were treated with SDS and DTT 1 M and the run was conducted at 40 mA. The samples were stained with Coomassie brilliant blue R-250. The SDS-PAGE profile of BTL-2 showed high molecular homogeneity in a single band with estimated molecular mass of approximately 9.0 kDa.

**Figure 2:**
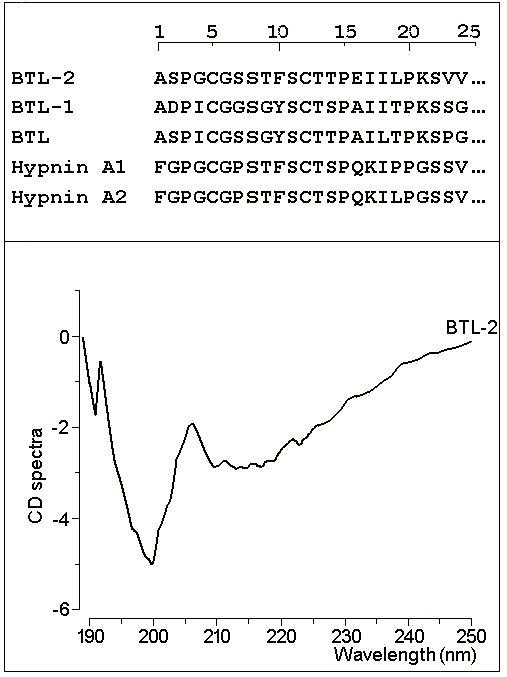
**2a) BTL-1 and BTL-2 were previously reduced and carboxymethylated and later sequenced in an automatic gas-liquid sequencer Procise f (Applied Biosytem).** 2b) The CD spectra were obtained using a J715 spectropolarimeter (Jarco Cop., Japan). BTL-2 was dissolved in water and the solution was adjusted to a final concentration of 10 mM of protein.

After the first purification step, on a preparative DEAE column, the main fraction, named *Bryothamnion triquetrum *lectin (BTL), was obtained and then dialyzed and lyophilised. The chromatography of BTL showed the presence of two main fractions, further named BTL-1 and BTL-2, which is the most abundant isoform isolated (Fig. 1a). After Reverse Phase HPLC, lectins were obtained with a high degree of purity (Fig. 1b). SDS-PAGE of BTL-2 revealed the presence of one main protein band with an estimated molecular mass of 9.0 kDa (Fig. 1c). The N-terminal sequences of both lectins were deduced and showed a high homology with BTL [20] and hypnins [19] (Fig. 2a). In addition, BTL-1 and BTL-2 had a pI of around 8.6. Circular dichroism spectra of BTL-2 were obtained in the wavelength region of 190 – 250 nm, revealing mostly random coil structures and a low content of α-helices or β-sheets (Fig. 2b).

**Figure 3:**
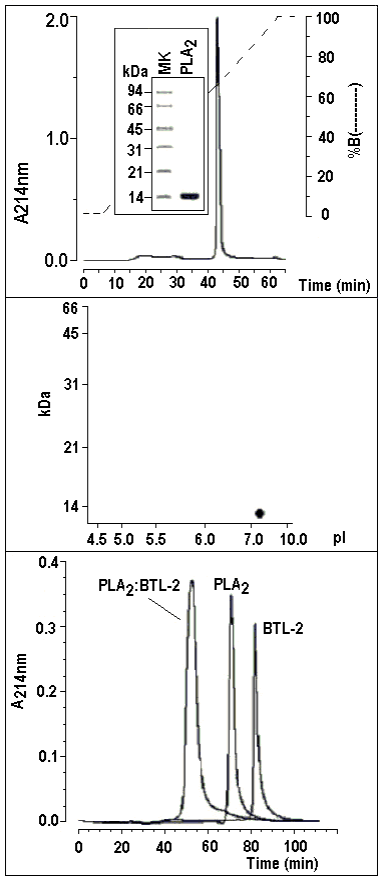
**3a) The venom from the *Crotalus durissus cascavella *rattlesnake was fractionated by HPLC molecular exclusion chromatography (Superdex 75 column 1 × 60 Cmm) and the active fraction was subjected to Reverse Phase HPLC (μ-Bondapack C18 column) to obtain a high purity protein.** 3b) Following the Laemmli method [43], a discontinuous electrophoresis was performed with a final acrylamide concentration of 12% in the running gel. All the samples and the molecular markers were treated with SDS and 1 M DTT and the run was conducted at 40 mA. The samples were stained with Coomassie brilliant blue R-250. The SDS-PAGE profile of PLA_2 _showed high molecular homogeneity in a single band with an estimated molecular mass of approximately 14.0 kDa. 3c) Two dimensional electrophoresis showing one protein spot with molecular mass of around 14 kDa and pI of approximately 8.3. 3d) One milligram of sample was dissolved in 0.5 mL of 0.05 M phosphate buffer, pH 7.5, and applied to a Protein Pack TSK gel 3000 column (0.8 Cmm 30 Cmm) equilibrated with this same buffer. This procedure was performed with one milligram of each protein, incubated together and with all molecular mass markers to estimate the molecular weight of the samples. It is possible to observe a peak with an estimated molecular mass of 10 kDa from BTL-2, another with 15 kDa from PLA_2 _and another of approximately 24–26 kDa that corresponds to both incubated proteins.

PLA_2 _was purified to high molecular homogeneity by reverse phase HPLC (Figure 3a), which resulted in only one electrophoretic band (Figure 3b). The estimated pl value of this purified protein was 8.3 (Figure 3c). Molecular exclusion chromatography indicated a molecular mass of approximately 10.0 kDa for the isolated BTL-2; 15.0 kDa for the isolated PLA_2_, while the PLA_2 _and BTL-2, when incubated for 30 minutes, had a single peak with a molecular mass of approximately 24.0 – 26.0 kDa (Figure 3d).

**Figure 4:**
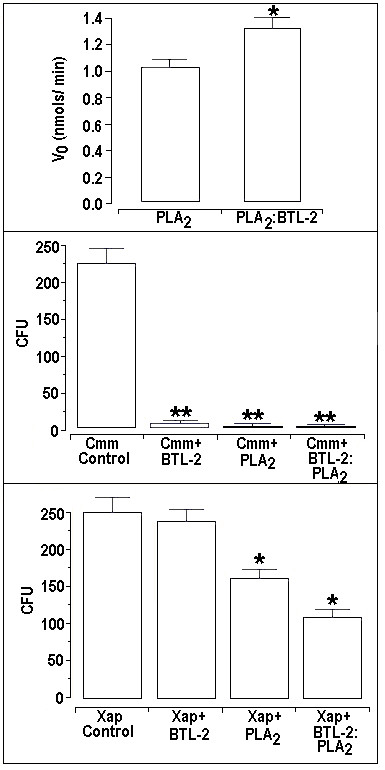
**4a) The enzymatic activity assay [45] was analyzed using an Elisa plate containing 200 μL of buffer (10 mM Tris-HCl, 10 mM CaCl_2_, 100 mM NaCl, pH 8.0), 20 μL of substrate (3 mM, 4-nitro-3-octanoyloxy-bencoic acid), 20 μL of water and 20 μL of the sample at a concentration 1 μg/μL or 2 μg/μL when both proteins were incubated together.** After 20 minutes from the beginning of the reaction, the absorbance was measured at 425 nm using a SpectraMax 340 multiwell plate reader (Molecular Devices). It is possible to observe that the enzymatic activity of the PLA_2 _was increased in the presence of BTL-2. 4b) and 4c) *Clavibacter michiganensis subsp. michiganensis *and *Xanthomonas axonopodis *pv.*passiflorae *were harvested from fresh agar plates and suspended in distilled and sterilized water (A600 nm = absorbance). BTL-2 demonstrated an inhibition of 98% of the growth of the Gram-positive bacterial strain, *Clavibacter michiganensis subsp. michiganensis *(Cmm), but only a 9.8% inhibition of the Gram-negative bacterial strain, *Xanthomonas axonopodis *pv *passiflorae *(Xap). PLA_2 _decreased Xap bacterial growth by 28.3% and Cmm growth by 98.5%, whilst incubation of these two proteins PLA_2_-BTL-2 inhibited their growths by 36.2% for Xap and 98.5% for Cmm.

The PLA_2 _was characterized as an enzymatically-active D49 PLA_2 _with moderate enzymatic activity in comparison with PLA_2 _from *Naja naja*. In the presence of BTL-2, we observed a significant increase of 23% in the enzymatic activity of PLA_2_, when compared with the PLA_2 _alone (Figure 4a).

BTL-2 demonstrated an inhibition of 98% in the growth of the Gram-positive bacterial strain, *Clavibacter michiganensis subsp. michiganensis *(Cmm), but only a 9.8% inhibition of the Gram-negative bacterial strain, *Xanthomonas axonopodis *pv *passiflorae *(Xap). PLA_2 _decreased Xap bacterial growth by 28.3% and Cmm growth by 98.5%, whilst incubation of these two proteins PLA_2_-BTL-2 inhibited their growths by 36.2% for Xap and 98.5% for Cmm (Figures 4b and 4c).

**Figure 5:**
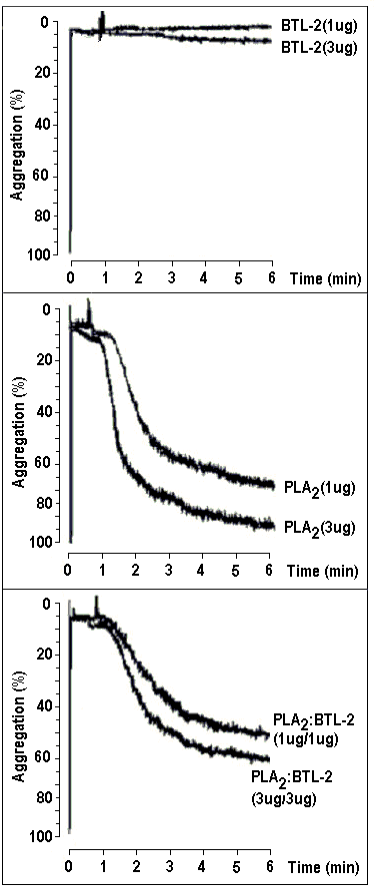
**The washed platelets assay was performed with venous blood collected from healthy volunteers.** The blood was centrifuged in 3.8% trisodium-citrate for 10 minutes at 200 × *g *and the residue was centrifuged again for 20 minutes at 1500 × *g*. Aggregations were performed in 1 μg and 3 μg concentrations in 20 μL of BTL-2, PLA_2 _and the two proteins (v/v). 5a) BTL-2 induced low aggregation, however the PLA_2 _induced high aggregation at these concentrations, and both proteins induced a lower aggregation when compared with the PLA_2 _alone.

**Figure 6:**
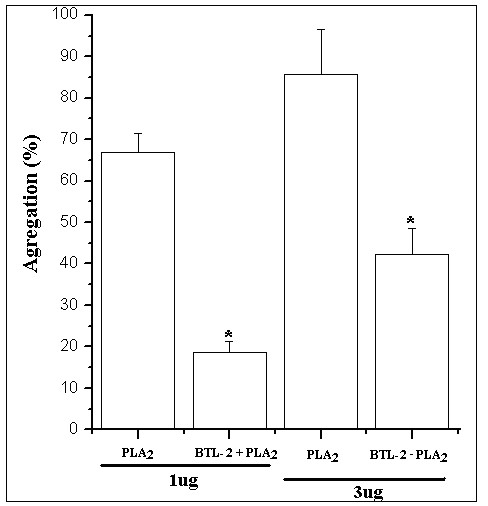
**6a) For the concentrations 1 μg and 3 μg, the PLA_2 _induced high aggregations.** When PLA_2 _was incubated for 30 minutes with BTL-2 at the same concentrations of 1 μg/1 μg and 3 μg/3 μg (v/v) in 20 μL volume it was possible to observe that the PLA_2 _induced a lower aggregation when compared to the PLA_2 _alone (n = 5; p < 0.05).

The PLA_2 _isolated from *Crotalus durissus cascavella *showed a strong platelet aggregation activity in washed platelets (Figures 5 and 6). The aggregation induced by PLA_2 _was very effective (Figure 5b), while BTL-2, at same concentration, did not produce any effect (Figure 5a). When PLA_2 _was incubated with BTL-2, we observed a significant decrease in the effect of PLA_2 _on platelet aggregation in the concentrations of 1 μg and 3 μg, respectivaly, 72.7% and 48.8%, when compared to PLA_2 _alone (Figure 5c). The averaged platelet aggregation assay data is depicted in Figure 6.

**Figure 7:**
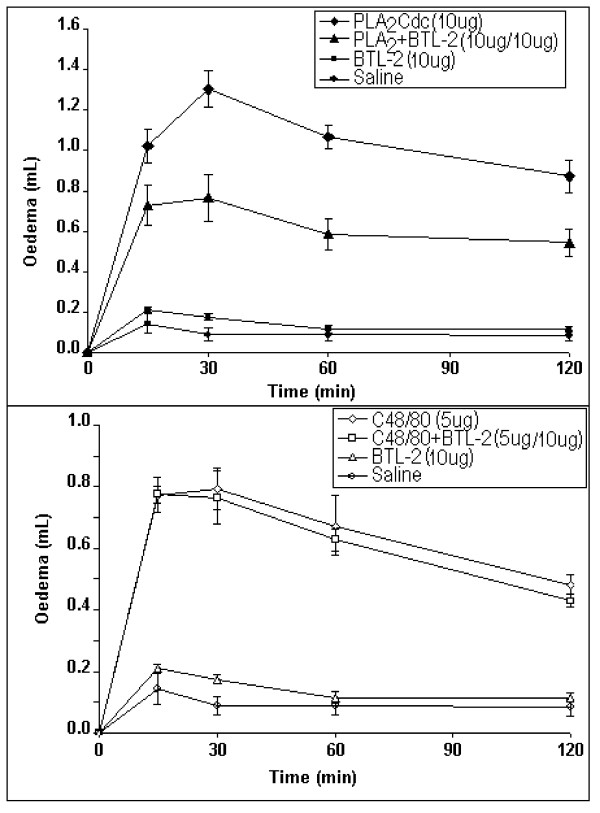
**Male Wistar rats (120 g–150 g) were anaesthetised with halothane (inhaled).** The paw oedema was induced by a single subplantar injection of 100 μL of sample 100 μg/paw). Paw volume was measured immediately before the injection and the intervals thereafter (15, 30, 60 and 120 minutes) using a hydroplethysmometer (model 7150, Ugo Basile, Italy). 7a) BTL-2 induced similar oedema simihat of the saline control. PLA_2 _showed the highest oedema at 30 minutes before the injection. The presence of BTL-2 decreased the oedema induced by the enzyme. 7b) The compound 48/80 (C48/80) was used, incubated with BTL-2, to show that the lectin was not acting on the tissues to decrease the action of PLA_2_, but was probably binding to the enzyme. Thus, the lectin did not decrease the action of C48/80 act.

BTL-2, injected in the paw of rats, did not induce an evident inflammation and its oedematogenic effect was similar to that of the saline control (Figure 7a). PLA_2 _from *C. durissus cascavella *venom induced a strong oedema response at 15 minutes following injection. The maximum inflammatory response was observed at 30 min following injection of PLA_2_. Under the same experimental conditions, PLA_2 _incubated with BTL-2, exhibited a lower oedematogenic effect, compared to non-incubated PLA_2 _(Figure 7a). In addition, BTL-2 did not inhibit the oedema induced by compound 48/80 (C48/80), a potent oedematogenic agent that induces the release of inflammatory mediators (Figure 7b).

**Figure 8:**
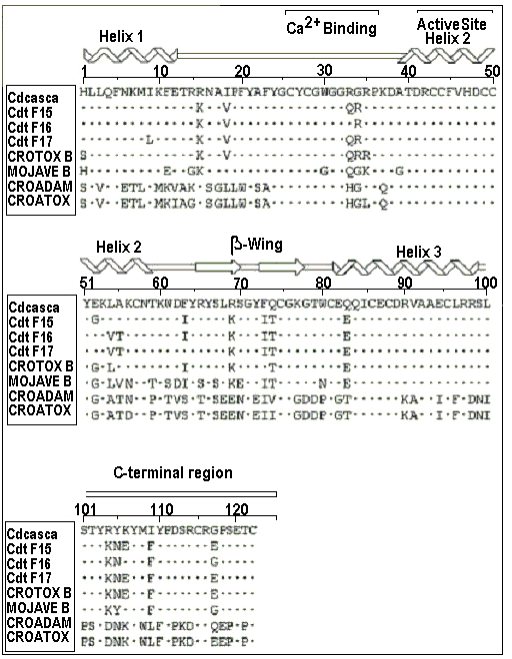
**Amino acid sequence of PLA_2 _isolated from *Crotalus durissus cascavella *and its estimated secondary structure.** The calcium binding loop, active site and C-terminal region are present in the sequence. Note the highly conserved calcium binding loop and active site, and an extensive portion of basic amino acid residues, such as Arg and Lys, in the C-terminal. This PLA_2 _showed high homology with other PLA_2_s, such as the isoforms from *Crotalus durissus terrificus *(Cdt F15, F16 and F17). Crotoxin B (cloned basic subunit of crotoxin) and mojave b (PLA_2 _subunit of *Crotalus scutulatus scutulatus*).

**Figure 9:**
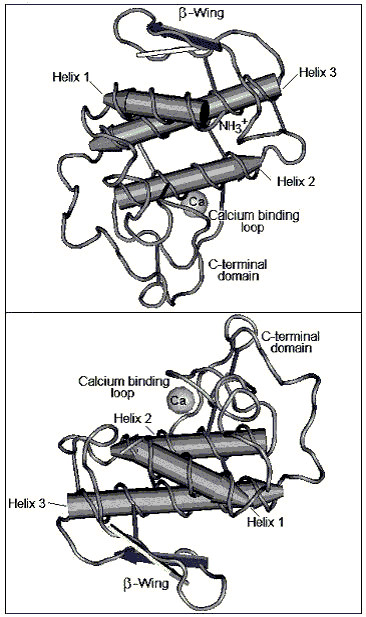
**The three dimensional model of PLA_2 _from *Crotalus durissus cascavella *venom was determined by comparative homology modelling using BLASTP in Protein Data Bank.** The model shows the β-wing, α-helix, calcium binding loop, and C-terminal loop.

PLA_2 _from *Crotalus durissus cascavella *shared a high degree of amino acid similarity with other rattlesnake venom PLA_2_s, such as the PLA_2_s isolated from *Crotalus durissus terrificus *isoforms 15, 16 and 17 (Cdt F15, F16 and F17), CROTOX B and MOJAVE B (Figure 8). Furthermore, the amino acid sequence of PLA_2 _was highly conserved at the N-terminal region, calcium-binding region, active site, α-helical and β-wing. Based on its three dimensional structure, *Crotalus durissus cascavella *PLA_2 _exhibited a high number of random coil structures, mainly in the calcium binding and the C-terminal loops (Figure 9a and 9b). The 3D model also suggests the presence of an extensive loop that connects helix 1 to helix 2 and other significantly long random coil structures on the C-terminal region (Figure 9a) in this PLA_2_.

## Discussion and Conclusion

The interaction between a marine alga lectin and a PLA_2 _from a rattlesnake was first detected by Hori et al 2000 [19]. Herein, we report the marine alga extract purification of BTL-2, one of the lectin isoforms isolated from the red marine alga *Bryothamnion triquetrum*. Lectins from *B. triquetrum*, with low molecular weight, share similar structural features with other low molecular weight lectins isolated from red algae, such as *Hypnea japonica *[19]. These structural similarities found suggest the importance of this protein for the development of these organisms and define a low molecular weight class of lectin.

Hori et al (2000) [19] suggested that the interaction between marine alga lectin and PLA_2 _was correlated to the presence of a specific interaction domain. Experiments conducted by these authors showed that the haemagglutination activity of hypnins was strongly inhibited by the addition of PLA_2_, which strongly suggests that the interaction between hypnins and PLA_2 _involves specific molecular recognition. Accordingly [19], hypnins have two distinct binding sites; a carbohydrate-recognition site(s) (probably containing a C-type CRD motif) and a protein-recognition site(s) (phospholipase A_2_-binding site). Interestingly, the interaction between PLA_2 _with hypnins did not affect the hemolytic activity induced by PLA_2_.

The N-terminal amino acid sequence of BTL-2 has a high homology with the lectins of *Hypnea japonica*, which probably possess the C-type CDR motif [19], as such BTL-2 probably possesses these same sites in its structure. The CD spectra analysis revealed that both BTL-2 and BTL-1 have a predominance of random coils, stabilized by disulfide bridges, conferring some interesting properties to these proteins such as resistance to high temperatures, very similar to hypnins which possess thermostability [19].

The catalysis of enzymatically-active PLA_2 _can be affected by factors such as substrate, and PLA_2 _activity is strongly enhanced by aggregated substrates that include micelles or monolayers [21]. Another important factor is substrate concentration, when the Critical Micelle Concentration (CMC) is reached, this enhances the enzymatic activity by several orders of magnitude [22,23]. Conformational changes in the structure of PLA_2 _are also an important factors that increase enzymatic activity [24]; these changes are located in the N-terminal residue and in the surface binding loop in which the calcium binding loop is located. The main conformation change, located in the N-terminal helix, has a small shift towards the calcium ion of PLA_2_. The role and structural relevance of the N-terminal region have also been reported [25-27]. In addition, the suppression or the chemical modification of the N-terminal region results in a strong reduction in the enzymatic activity of catalytically-active PLA_2_.

It has been reported that highly-purified heparin sodium salt is able to increase the enzymatic activity of catalytically-isolated PLA_2 _from *Crotalus durissus terrificus *venom [28,29]. The potentialization of the enzymatic activity of heparin has been observed in the treatment of some vascular disorders in clinical trials. On the other hand, the inhibitory effects of the neurotoxic or other pharmacological activities of PLA_2 _are well known. The mechanism of action of PLA_2 _towards heparin involves inducible conformation changes to the active site, the N-terminal region, and strong binding with the cationic site located in the C-terminal region [30-32], which leads to the modifications in the biological effects induced by the catalytically-active PLA_2_.

Our results showed that this novel basic hypnin-like lectin, isolated from *Bryothamnion triquetrum*, seems to form a heterodimeric structure with PLA_2 _from the *Crotalus durissus cascavella *venom and that its molecular binding strongly increases the enzymatic activity of PLA_2_. Furthermore, our data suggest that the complex that is established between PLA_2_, from *Crotalus durissus cascavella*, and lectin, from *Bryothamnion triquetrum*, probably involves a hydrophobic contact and indirectly affects the catalytic site of PLA_2_. Thus, our results show that some pharmacological activities, induced by PLA_2 _isolated from *Crotalus durissus cascavella*, did not involve its solely enzymatic activity, indicating that the specific actions induced by this PLA_2 _are due to the presence of pharmacological sites on the protein surface, which are distinct from catalytic sites, allowing PLA_2 _to bind specifically to soluble proteins or membrane-bound proteins that participate in the mechanism of action and this is not an isolated case, as observed for other PLA_2 _from other sources [33,34]. The antibacterial activity, however, is very dependent on the ability of this PLA_2 _to destroy the bacterial membrane. These facts are supported by the results of the bacterial growth rate of Xap, which was strongly inhibited by a complex formed by PLA_2 _and BTL-2.

Conversely, the platelet aggregation assay showed that the presence of the lectin significantly decreased the PLA_2 _aggregation effect, showing that the pharmacological activity of the enzyme was affected by BTL-2. This same effect occurred in the oedema assay, where in the presence of the lectin, the pharmacological activity of the PLA_2 _was decreased, inducing a lower oedema. The hydrolysis of arachidonic acid (AA) by the PLA_2 _generates free AA and lysophospholipids that are the precursors of eicosanoids and platelet-activating factor, respectively [35]. At first, it was thought that secretory PLA_2_s served to provide substrates for the biosynthesis of proinflammatory lipid mediators; however, it was found that not all PLA_2 _hydrolyze AA from intact mammalian cells, suggesting that the generation of lipid mediators is not a general function of this enzyme [36,37]. A great breakthrough was the identification of other biological effects of PLA_2 _in cells involved in inflammatory and immune responses. As such, these results support the idea of the presence of another pharmacological site in the PLA_2_, isolated from the *Crotalus durissus terrificus*, which is distinct from the enzymatic site. Something similar is observed in some other PLA_2 _that interact with target cells via membrane peptidoglycans and with specific or promiscuous receptors [11] and, in some cases, lead to activation of some intracellular cytoplasmic PLA_2 _and several proteins including PKA and PKC [37-39]. Some specific mutation studies carried out with PLA_2 _showed that the binding of secretory PLA_2 _to the receptor involves a specific recognition of the CRD region of this receptor [34,36,37], which is located near the catalytic site of PLA_2_. Our results, however, suggest that the PLA_2 _region involved in the binding with BTL-2 produces a structural change that results in the reduction of its pharmacological action, compared to untreated PLA_2_. The C48/80 experiment supports this argument, since it shows that the lectin was not binding to the tissues to hinder the C48/80 or PLA_2 _activity, but that it was acting in the PLA_2_.

Class I and II secretory PLA_2_s possess an antiparallel, poorly conserved β-wing. The structural conformation, though variable in relation to the rest of the enzyme, is internally consistent across species. The wing extends outwards into the solvent, and the 'wingtip' is poorly anchored. It has been suggested that biological properties such as neurotoxicity, myotoxicity or anticoagulation may be associated with the β-wing [40-42]. The presence of a similar β-wing motif in the 3D model of the PLA_2_, isolated from *Crotalus durissus cascavella *venom, may explain its remaining pharmacological activity, which probably has two distinct pharmacological sites, one located in the random coil that links the N-terminal helix with the helix 2 and the β-wing.

The main conclusions of this study are that; 1) the pharmacological activity of PLA_2 _from *Crotalus durissus cascavella *is completely dissociated from its enzymatic activity and also some of its biological activity. 2) The PLA_2 _model revealed that the presence of an extensive hydrophobic random coiled region may be involved in the interaction with BLT-2, forming a random-coil based structure.

## Methods

### Source of algae

Specimens of the red marine alga *Bryothamnion triquetrum *were collected from the Atlantic Coast of Brazil (Pacheco Beach, Ceará) and kept in plastic bags at -20°C until processed.

### Lectin purification

Algae were thawed, rinsed with distilled water, cleaned of epiphytes, and ground to a fine powder under liquid nitrogen. The powder was stirred for 4 hours with 0.02 M phosphate buffer (pH 7.0), with 0.15 M NaCl, filtered through nylon tissue, and then centrifuged at 7,000 × *g*, for 30 min, at 4°C. The supernatant was acidified and remained under refrigeration for 4 hours. The precipitated pigments were removed by centrifugation and the supernatant (crude extract) was adjusted to pH 7.0, followed by ammonium sulphate precipitation (0–60%). Precipitated proteins were recovered by centrifugation, dialyzed in distilled water and applied onto a DEAE-cellulose column, equilibrated and eluted with 0.02 M phosphate buffer (pH 7.6), followed by elution with 1.0 M NaCl solution in the same buffer, at a flow rate of 30 mL/h. The unabsorbed fractions with haemagglutinating activity were pooled and rechromatographed in the same column. Active fractions containing lectins were dialyzed and lyophilised (DEAE fraction).

After that, approximately 5 mg of freeze-dried DEAE-fraction was dissolved in 250 μL of ammonium bicarbonate buffer (0.05 M; pH 7.9) and homogenised until complete dissolution, followed by clarification by centrifugation (4,500 × *g *for 2 min, at 27°C). The supernatant was recovered and injected onto an ion exchange HPLC column (Protein Pack SP 5PW, 0.75 × 10 cm, Waters), previously equilibrated with the same buffer used for dissolving the fraction. The elution was conducted using a discontinuous linear gradient of ammonium bicarbonate at a concentration of 1.0 M, pH 8.0, at constant flow rate of 1 mL/min.

The chromatography was monitored at 280 nm and the peaks were collected and the two fractions containing haemagglutinanting activity were lyophilised and than subjected to the final chromatographic steps of the Reverse Phase (RP-HPLC). The elution of samples was carried out using a linear gradient concentration of an aqueous solution of 66% acetonitrile. Samples were previously dissolved in buffer A of RP-HPLC (0.1% TFA) that was used to equilibrate the column. The fractions were immediately concentrated by ultrafiltration (AMICON), followed by dialysis with 5.0 mM ammonium bicarbonate solution (pH 7.8), sterilized by microfiltration (0.22 μm) and stored at 4°C for further use.

### Electrophoresis

The electrophoresis was carried out following the Laemmli method [43]. The degree of purity of fractions was assessed by discontinuous electrophoresis using a final acrylamide concentration of 12% in the running gels (1.0 M Tris-HCl, pH 8.8) and 5% in the stack gel (0.5 M Tris-HCl, pH 6.8). The electrophoretic run was carried out using a BIORAD electrophoretic system for SDS-PAGE. All samples and the molecular marker were treated with SDS and 1.0 M DTT and the run was conducted at 40 mA for both plates. After electrophoresis, samples were stained with Coomassie brilliant blue R-250.

### Circular dichroism

The purified BTL-2 was dissolved in water and the solution was adjusted to a final concentration of 10 mM of protein. The sample was transferred to a 1 mm path-length quartz cuvette. Circular dichroism spectra in the wavelength range of 185–300 nm were obtained using a J715 spectropolarimeter (Jasco Corp., Japan) using a bandwidth of 1 nm and a response time of 1s. Data collection was performed at room temperature with a scanning speed of 100 nm/min; a total of nine scans were accumulated for the sample.

### PLA_2 _purification

PLA_2 _from the rattlesnake, *Crotalus durissussus casvella*, was purified by a combination of chromatographic procedures and its molecular homogeneity was determined by SDS-PAGE. The whole venom was fractionated by HPLC molecular exclusion chromatography using a Superdex 75 column (1 × 60 cm). The active PLA_2 _fraction was then subjected to Reverse Phase HPLC in a C18 μ-Bondapack column (0.78 cm × 30 cm) (Waters 991-PDA system). The column was eluted with a linear gradient (0–66.5%, v/v) of acetonitrile (solvent B) at a flow rate of 2 mL/min, and absorbance was monitored at 280 nm. The fractions were collected manually, lyophilized and stored at -20°C.

### Amino acid sequence

Three milligrams of purified protein were dissolved in 200 μl of 6 M guanidine chloride (Merck, Darmstadt, Germany) containing 0.4 mM Tris-HCl and 2 mM EDTA (final pH 8.15). Nitrogen was flushed over the top of the protein solution for 15 min, which was then reduced with DTT (6 M, 200 μl) and carboxymethylated with ^14^C-iodoacetic acid and cold iodoacetic acid. Nitrogen was again flushed over the surface of the solution and the reaction tube sealed. This solution was incubated in the dark at 37°C for 1 h and desalting was performed on a Sephadex G 25 column (0.7 × 12 cm) in 1 mM acetic acid buffer. The eluted reduced and carboxymethylated (RC) protein was lyophilised and stored at -20°C. The RC lectins were then purified and the N-terminal region sequenced.

For the RC-PLA_2_, we determined the complete amino acid sequence of the protein by sequencing the N-terminal region and the peptides from enzyme-treated PLA_2_. One sample of RC-protein was digested with *Staphylococcus aureus *protease V8 for 16 hours at 37°C, using an enzyme -substrate ratio of 1:30. The reaction was stopped by lyophilisation. A second sample of RC-PLA_2 _was digested with Clostripain for 8 hours at 37°C and then lyophilised [44]; the products were separated by Reverse Phase HPLC using a Waters PDA 991 system with a C-18 μ-Bondapack column.

Peptide peaks were isolated using a linear gradient (0–100% of acetonitrile in 0.1% TFA (V/V)). A third sample (2 mg) was cleaved with a 15-fold molar excess of cyanogens bromide (CNBr) over methionine residues in 70% formic acid (4 ml) under nitrogen for 24 hours at room temperature. The reaction mixture was then diluted with 40 mL of water and lyophilised. Excess reagents were removed by gel filtration on a Sephadex G-25 column (1 × 20 cm) equilibrated with 10% acetic acid. The CNBr peptide fragments were separated by Reverse Phase HPLC, using an analytical μ-Bondapack C18 column (0.39 × 30 cm; Waters) with 0.1% TFA as solvent A and acetonitrile containing 30% of solvent A (solvent B). The elution profile was monitored at 214 nm. Analysis of the amino acid sequence of the RC-protein, as well as that of the enzymatically-digested fragments were performed with an Applied Biosystems model Procise f gas-liquid protein sequencer. The phenylthiohydantoin (PTH) derivatives of the amino acids were identified with an Applied Biosystems model 450 microgradient PTH-analyser.

### PLA_2 _activity

PLA_2 _activity was measured [45]. The standard assay mixture contained 200 μL of buffer (10 mM Tris-HCl, 10 mM CaCl_2_, 100 mM NaCl, pH 8.0), 20 μL of 2.98 mM substrate (4-nitro-3-octanoyloxy-benzoic acid; 4N_3_OBA), 20 μL of water, and 20 μL of PLA_2 _(1 mg/mL) in a final volume of 260 μL. After the addition of PLA_2_, the mixture was incubated for 20 min at 37°C. The increase in absorbance at 425 nm, due to product formation, was monitored during this period and the activity was expressed as nmols of product formed nmols/min/mg. The inhibitory effect of BTL-2 (1 mg/mL) on PLA_2 _activity was investigated by incubating the two proteins at 37°C for 30 min prior to assaying the enzyme activity using the SpectraMax 340 multiwell plate reader (Molecular Devices).

### HPLC molecular exclusion chromatography of BTL-2 andBTL-2-PLA_2_

One milligram of PLA_2 _from *C. d. cascavella *was dissolved in 0.5 mL of 0.05 M phosphate buffer, pH 7.5, and applied to a Protein-Pack TSK gel 3000 column (0.8 cm × 30 cm), previously equilibrated with the same buffer before use. BTL-2 and PLA_2 _(1:1) were preincubated together for 30 minutes in phosphate buffer. Separately, molecular mass markers (bovine serum albumin, 66 kDa; egg album, 45 kDa; carbonic anhydrase, 29 kDa, and lysozyme 14 kDa) were used to estimate the molecular weight of PLA_2_, BTL-2 and PLA_2_:BTL-2. All molecular weight markers were dissolved in phosphate buffer at a concentration of 2 mg/mL. The proteins were eluted at a flow rate of 0.2 mL/min and the absorbance profile was monitored at 214 nm.

### Platelet aggregation studies

#### Sample collection and aggregation studies

Venous blood was collected with informed consent from healthy volunteers who denied taking any medication in the previous 14 days. Blood was collected by a two-syringe technique using polypropylene syringes and 19-gauge needles, and immediately transferred into polypropylene tubes containing 1/10 of final volume of 3.8% trisodium citrate.

After removing the platelet-rich plasma (PRP), the remaining blood was prepared by centrifugation of citrated blood at 200 × *g *for 10 min and washed platelets (WP) were obtained from the residue by centrifugation of citrated blood at 1500 × *g *for 20 min. The platelets were left for 1 h at room temperature to recover their sensitivity to aggregating agents. Platelet counts were performed on a Coulter S Plus (Coulter Electronics, Hialeah, FL) or by phase-contrast microscopy. Platelet aggregation was carried out using 400 μL of the washed platelets solution in a cuvette and kept at 37°C with constant stirring.

The desired concentration of protein was added 3 minutes prior to the addition of platelet aggregation inducers (thrombin for washed platelets). Subsequently, the aggregation was recorded for 5–10 min by using an aggregometer (Payton Scientific Instruments, Inc, Buffalo, NY). Aggregation experiments were performed in two concentrations (1 μg and 3 μg) of BTL-2, PLA_2_, and PLA_2 _incubated with BTL-2 (v/v) in a final volume of 20 μL, which was previously incubated (30 min) at room temperature.

### Paw oedema assay

Male Wistar rats (120–150 g) were anaesthetised with halothane (inhaled). The rat paw oedema was induced by a single subplantar injection of 100 μL of BTL-2 (10 μg/paw), PLA_2 _(10 μg/paw), and PLA_2 _incubated with BTL-2 (v/v). Paw volume was measured immediately before the injection of the samples and at selected time intervals thereafter (15, 30, 60, and 120 minutes) using a hydroplethysmometer (model 7150, Ugo Basile, Italy). The compound 48/80 (C48/80) was incubated with BTL-D2 to show that the lectin was not acting on the tissues to decrease the action of the PLA_2_. All samples were dissolved in sterile saline solution (0.9%). Results were expressed as the increase in paw volume (mL) and calculated by subtracting the basal volume.

### Antibacterial assay

*Xanthomonas axonopodis *pv.*passiflorae *(Xap) and *Clavibacter michiganensis subsp. michiganensis *(Cmm) were harvested from fresh agar plates and suspended in distilled and sterilized water (A600 nm = absorbance, corresponding to 3 × 10^8 ^CFU/mL). Aliquots of this suspension were diluted to 3 × 10^3 ^CFU/mL and incubated with 100 μL of BTL-2 (1 mg/mL) for 30 min at 37°C. We then determined the survival on Agar Nutrient (Difco) Plate (n = 5).

### Prediction of the three-dimensional structure of PLA_2_

Amino acid sequence similarity and alignment searches were carried out to compare the PLA_2 _isolated from *Crotalus durissus cascavella *venom against the Protein Data Bank using BLASTP. The 3D structure of PLA_2 _was determined by comparative homology modelling. Using computer graphics, the amino acid sequence of PLA_2 _was fitted to the high-resolution X-ray structure of the homologous 1BJJ_A. This allowed the construction of a model, which was then subjected to energy minimization and molecular dynamic simulations.

### Statistical analyses

The results were reported as the means ± SEM of *n *experiments. The significance of differences between means was assessed by analysis of variance, followed by a Dunnett's test when several experimental groups were compared with the control group. The confidence limit for significance was 5% (p > 0.05).

## Authors' contributions

SCBO -carried out all the purifications, activity of the PLA_2_, eletrophoresis, participated in all the assays, coordination of the study, helped to draft the manuscript and performed the statistical analysis. FVF; EA; EAC; RPM – carried out the pharmacological assays e.g. platelet aggregation studies and the paw oedema assay. RA – carried out the circular dichroism.

DOT; LOSB – carried out the antibacterial assays. EVN; BSC; CSN; AHS; KSN -collected the marine alga and performed the initial purification of the alga lectin. MHT – carried out the amino acid sequencing, prediction of the three-dimensional structure of PLA_2_, coordination of the study, helped to draft the manuscript and performed the statistical analysis. All authors read and approved the final manuscript.
